# Polydatin Encapsulated Poly [Lactic-co-glycolic acid] Nanoformulation Counteract the 7,12-Dimethylbenz[a] Anthracene Mediated Experimental Carcinogenesis through the Inhibition of Cell Proliferation

**DOI:** 10.3390/antiox8090375

**Published:** 2019-09-05

**Authors:** Sankaran Vijayalakshmi, Arokia Vijaya Anand Mariadoss, Vinayagam Ramachandran, Vijayakumar Shalini, Balupillai Agilan, Casimeer C. Sangeetha, Periyasamy Balu, Venkata Subbaih Kotakadi, Venkatachalam Karthikkumar, David Ernest

**Affiliations:** 1 Department of Biotechnology, Thiruvalluvar University, Serkadu, Vellore 632 115, Tamilnadu, India; 2 Department of Physics, Sri Padmavati Mahila Visvavidyalayam, Tirupati 517502, Andra Pradesh, India; 3 Department of Chemistry, Thiruvalluvar University, Serkadu, Vellore 632 115, Tamilnadu, India; 4 DST-PURSE Center, Sri Venkateswara University, Tirupathi 517 502, Andhra Pradesh, India; 5 Department of Pharmacology and Therapeutics, College of Medicine and Health Sciences, UAE University, Al Ain 17666, UAE

**Keywords:** polydatin, PLGA, nanoformulation, antioxidant, cell proliferation, apoptosis

## Abstract

In the present study, the authors have attempted to fabricate Polydatin encapsulated Poly [lactic-co-glycolic acid] (POL-PLGA-NPs) to counteract 7,12-dimethyl benzyl anthracene (DMBA) promoted buccal pouch carcinogenesis in experimental animals. The bio-formulated POL-PLGA-NPs were characterized by dynamic light scattering (DLS), Fourier transform infrared (FTIR) spectroscopy, X-ray powder diffraction (XRD) pattern analysis, and transmission electron microscope (TEM). In addition, the nano-chemopreventive potential of POL-PLGA-NPs was assessed by scrutinizing the neoplastic incidence and analyzing the status of lipid peroxidation, antioxidants, phase I, phase II detoxification status, and histopathological changes and in DMBA-treated animals. In golden Syrian hamsters, oral squamous cell carcinoma (OSCC) was generated by painting with 0.5% DMBA in liquid paraffin three times a week for 14 weeks. After 100% tumor formation was observed, high tumor volume, tumor burden, and altered levels of biochemical status were observed in the DMBA-painted hamsters. Intra-gastric administration of varying concentration of POL-PLGA-NPs (7.5, 15, and 30 mg/kg b.wt) to DMBA-treated hamsters assumedly prevents oncological incidences and restores the status of the biochemical markers. It also significantly enhances the apoptotic associated and inhibits the cancer cell proliferative markers expression (p53, Bax, Bcl-2, cleaved caspase 3, cyclin-D1). The present study reveals that POL-PLGA-NPs is a penitential candidate for nano-chemopreventive, anti-lipid peroxidative, and antioxidant potential, and also has a modulating effect on the phase I and Phase II detoxification system, which is associated with reduced cell proliferation and induced apoptosis in experimental oral carcinogenesis.

## 1. Introduction

Cancer has a high mortality rate, and around 18.1 million people are diagnosed with cancer each year. According to the World Health Organization (WHO) statistics, by 2030, this number will be almost double [1], and a recent report from the Indian council of Medical Research Council states that by 2020, 1.73 million new cancer cases will be detected, and over 8.8 lakh deaths will occur due to cancer [2]. The drastic incidence and mortality rate of cancer is associated with age, sex, and race. Risk factors for cancer incidence include (i) tobacco smoking, which causes lung, head, and neck cancer; (ii) drinking alcohol, which causes liver, esophageal, breast, oral and other cancers; (iii) physical inactivity; and (iv) a diet low in fruit and vegetables, which can increase the risk of colon, breast, and possibly other types of cancers [3,4].

The conventional therapeutic management of cancer, e.g., surgery, chemotherapy, radiation, and hormonal therapy, are still ineffective for the management of cancer progression. Hence, less toxic and more effective anti-cancer agents for the management of cancer are urgently needed. Polydatin (C_20_H_22_O_8_) is a monocrystalline glycisidic phyto-compound found in Sitka spruce, grape, peanut, hop cones, red wines, hop pellets, and cocoa [5]. Pharmacological and clinical studies have revealed that polydatin has anti-arteriosclerosis, anti-tumor, anti-oxidative, anti-inflammatory, anti-proliferative, anti-angiogenic, hepatoprotective, and immunoregulatory effects. In recent times, the cancer preventive potential of polydatin has also been examined. It act as repressor candidate of tumorogenesis, through the hindrance of cell proliferation, invasion, migration, and induced cell apoptosis [6]. In addition, Chen et al. (2017) have reported that polydatin suppressed the cell cycle progression and enhanced the apoptotic associated gene expression in human cancer cell lines [7]. It also suppresses the breast carcinogenesis in MCF-7 cells and gradually down-regulates the expression of phosphor-NF-κB p65 and activation of NF-κB pathway in non-small cell lung cancer [8]. In a recent study, Hu et al. (2018) suggested that polydatin modulated the VEGF-induced angiogenesis by suppressing the phosporylatin of Akt, eNOS, and Erk [9]. In addition, it induces autophagy and apoptosis in multiple myeloma cells through the inhibition of mTOR/p70s6k pathway [10].

Biodegradable polymeric agents have been extensively used to improve the bioavailability of plant based chemotherapeutic agents. Considering this, we utilized the poly-lactic-co-glycolic acid (PLGA) for the synthesis of polydatin nanoparticles. PLGA is one of the most extensively used biodegradable polymers because its hydrolysis leads to endogenous and easily metabolized monomers of lactic acid and glycolic acid [11]. Recent publications suggest that PLGA-NPs functionalized with (i) ß-Sitosterol, a natural phytosterol, (ii) resveratrol, a natural polyphenol, and (iii) tea polyphenols of theaflavin and epigallocatechin-3-gallate [12,13,14] might be potential candidates for cancer treatment. Many studies have revealed that encapsulation of PLGA nanoparticles improves the biocompatibility, tunable mechanical property, and controllable degradation of several chemotherapeutic drugs including paclitaxel, tamoxifen, and anthracyclines. Wang et al. (2014) used a soy-phospholipid based liposome system to improve the solubility and bioavailability of polydatin [15]. Yallapu et al. (2010) documented the nano-formulation of PLGA improve the therapeutical efficacy of curcumin in human ovarian and metastatic breast cancer cell lines [16]. Since, there are no reports are available the combinational physiochemical features of polydatin loaded PLGA nanoparticles. To the best of our knowledge, this is the first report on the biosynthesis of Polydatin-loaded PLGA nanoparticles (POL-PLGA-NPs). The findings of this study validate that the high negatively charged synthesized nanoparticles have the ability to penetrate into inside the tumor cells via sustainable drug releasing profile. It could be promising. The feasible outcome of this study, hopefully provide new insights of nanochemopreventive potential POL-PLGA-NPs.

Hence, the aim of the study is to employ a simple method for the bio-fabrication of POL-PLGA-NPs. The efficiency of the structural modification of POL-PLGA-NPs was evaluated by FTIR and FRD analysis. The physical-chemical characteristics, namely, average size, morphological features, zeta potential, drug loading efficiency, and encapsulation efficacy, were determined. Further, the apoptotic activating efficacy of POL-PLGA-NPs in DMBA induced buccal pouch carcinogenesis was investigated.

## 2. Materials and Methods

### 2.1. Chemicals and Reagents

DMBA, PLGA (lactide: glycolide 75:25, Mw 76,000–115,000), polydatin and bovine serum albumin (BSA) were obtained from Sigma-Aldrich Chemical (St. Louis, MO, USA). Primary antibodies, such as mutant p53, Bax, Bcl-2, cleaved caspase 3, cyclin-DI and ß-actin were purchased from Santa Cruz Biotechnology (Santa Cruz, CA, USA). All other chemicals and solvents were supplied from Himedia laboratories, Mumbai, India and Fisher Inorganic and aromatic Limited (Chennai, India).

### 2.2. Synthesis of Polydatin Encapsulated PLGA Nanoparticles [POL-PLGA-NPs]

Polydatin encapsulated PLGA nanoparticles [POL-PLGA-NPs] were fabricated by an oil/water emulsion method with minor modification [17]. 50 mg of PLGA was dissolved in 5 mL of dichloromethane and acetone (prepared as 3:2) to form well-proportioned PLGA solution in a round-bottomed flask. Then, 10 mg of polydatin was added to the solution and sonicated at 200 W for 10 min to make a primary emulsion (organic phase) and the resultant primary emulsion was added dropwise to BSA solution (1% *w/v*) (aqueous phase) and the mixture was sonicated at 200 W for 15 min to make an oil/water (O/W) emulsion. To diffuse the O/W emulsion, 15 mL of deionized water was added and stirred vigorously to eliminate the residual organic solvent. After continuous stirring for 2–3 h, the solution was centrifuged at 14,000 rpm for 30 min, the supernatant was discharged and the pellet was washed repeatedly with deionized water. After the centrifugation of 10,000 rpm for 20 min, the POL-PLGA-NPs which settled down was collected and lyophilized by freeze drying and stored at 4 °C.

### 2.3. Characterization of Nanoparticles

After the successful synthesis of Polydatin-encapsulated PLGA nanoparticles were processed for physicochemical characterization techniques. Particle size, polydispersity index and zeta potential of PLGA-NPS/POL-PLGA-NPs was investigated by dynamic light scattering (DLS) using Horiba Scientific-SZ-100 (Horiba, Kyoto, Japan). X-ray diffraction pattern (XRD) of the crystalline phase was recorded using an Ultima IV X-ray diffractometer (X’pert-pro MPD-PANalytical, Netherland) at the angle range of 2θ (10–80°). Surface chemistry of the nanoparticles and functional group analysis was done by Fourier transfer infrared spectroscopy (FTIR) (FTIR PerkinElmer Paragon 500, USA). Particle size and topological features of the nanoparticles were recorded by Transmission electron microscope using Philips CM120 M (80 kV; Philips, Eindhoven, Netherlands) and the three-dimensional features of the individual and the groups of particles are investigated by atomic force microscope (AFM) using AFM-Solver Next (NT-MDT, Moscow, Russia).

### 2.4. Determination of Encapsulation and Loading Efficiency of POL-PLGA-NPs

The encapsulation efficiency (EE) and drug loading efficiency (LE) of POL-PLGA-NPs were determined by spectrophotometric method. Briefly, 3 mg of POL-PLGA-NPs was dissolved in 6 mL of PBS and centrifuged at 12,000 rpm for 30 min. The content of free polydatin in the supernatant was measured by UV-Vis spectrophotometer (Elico SL 196, Hyderabad, India) at 230 nm. The percentage of EE and LE was calculated from this equation:

EE (%) = W_0_/W_1_ × 100; LE (%) = W_0_/W × 100 (1)

Here, W_0_ is the amount of polydatin enveloped in the PLGA nanoparticles, W is the amount of polydatin encapsulated nanoparticles, and W_1_ is the amount of polydatin added in the system.

### 2.5. In Vitro Releasing Profile of Polydatin

The amounts of polydatin released from the polydatin-encapsulated PLGA nanoparticles were determined by the spectroscopic method using dialysis bag. In this study, we were chosen for two different pH of 4.8 and 7.4 to simulate the extracellular and lysosomal environment, respectively. In briefly, 10 mg of sample were immersed in a dialysis bag and flooded into 50 mL of phosphate buffer saline (PBS) at different pH (4.8 and 7.4) at under constant and continuous shaking (100 rpm at 37˚C). At the scheduled time intervals (2, 4, 8, 12, 24, 48 h), samples were taken from the solution and the volume was replaced with fresh PBS and the released content of polydatin was measured by UV-visible spectrophotometer (Elico SL 196, Hyderabad, India).

### 2.6. Animals

Eight- to 10-week-old Syrian hamsters weighing 90–120 g were obtained from Indian Council of Medical Research (ICMR)-National Animal Resource Facility for Bio-Medical Research (NARFBR), Hyderabad, India. The animals were housed in ventilated cages under the constant conditions (22 °C, 12 h light/dark cycle). The animals were fed with a normal pellet diet (Hindustan Lever Ltd., India) and water ad libitum. Animals’ care, experimental procedure, and euthanasia procedure was performed by the guidelines of the committee for the purpose of control and supervision on experiments on animals (CPCSEA) and the protocol was approved by the institutional ethical committee (1282/PO/Re/S/09/CPCSEA).

### 2.7. Treatment Protocol

After allowing the animals one week of acclimation to their new environmental conditions, they were randomized into control and experimental groups and separated into six groups (*n* = 6 animals). Group 1 animals served as control. The animals in the groups (2–5) were painted with 0.5% solution of DMBA in mineral oil using number 4 hair brushes to induce oral carcinogenesis. Every application giving 0.4 mg DMBA load. Carcinogenic control animals had not received any other treatment (Group-2). Groups of 3–5 animals (Nanoparticles treated group) were orally treated with (intra-gastric mode-infant feeding tube No: 5) different concentrations of POL-PLGA-NPs (7.5, 15, and 30 mg/kg b.wt; dissolved in 0.2% DMSO) by intragastric intubation thrice a week on alternate days of the DMBA application. Groups of 6 animals were orally administrated with 30 mg kg/b.wt of POL-PLGA-NPS to check its adverse effects. Vehicle control animals were painted with liquid paraffin throughout the study (Group-1). After the treatment schedule, the animals were sacrificed; blood, liver, and buccal pouches were used to biochemical, histopathological, and molecular studies. The body weights of all hamsters were recorded until the end of the experiment. Tumor incidence, tumor weight and tumor volume were accessed by the method of Geren et al. [18].

### 2.8. Histological Studies

Part of the buccal tissue was surgically removed and immersed in 10% formalin for 24 h for fixation. Then the tissue was processed and embedded in paraffin wax, 4–5 µm sections were sliced and stained with hematoxylin and eosin. The sections were examined under a light microscope and photo-micrograph was documented.

### 2.9. Biochemical Estimations

The tissue lipid peroxidative byproducts known as thiobarbituric acid reactive substances (TBARS) level was measured as described by Ohkawa et al., and the formation of the pink-colored chromogen was measured at 532 nm [19]. Lipid hydroperoxides (LOOH) content was estimated by the method of Jiang et al. [20] and the Conjugated dienes (CD) levels was measured by the method of Rao and Recknagel [21]. Superoxide dismutase (SOD, EC.1.15.1.1) activity was estimated by the method of Kakkar et al., and the percentage of inhibition of formazan development was calculated. The amount of enzyme required for 50% inhibition of NBT reduction/min/mg protein defined as one unit of the enzyme [22]. Catalase (CAT, EC.1.11.16) activity was assayed by the method of Sinha. The reaction of tissue homogenate with H_2_O_2_, the presence of buffer was arrested by the addition of a dichromate acetic acid reagent, and the formation of chromic acetate was measured at 590 nm [23]. The values are expressed as µmoles of H_2_O_2_ utilized/min/mg protein. The levels of reduced glutathione (GSH) was assessed by the method of Ellman, which is based on the reduction of 5, 5′ dithiobis 2-nitrobenzoic acid (DTNB) and the glutathione concentration was expressed as µmoles of -SH content/g tissue [24]. Vitamin E level was measured by the method of Palan et al., This method incorporates the reduction of Fe^3+^ to Fe^2+^ by a-tocopherol and the formation of a colored complex was measured at 520 nm [25]. Vitamin C level was estimated by the method of Omaye et al., This method involves the oxidation of ascorbic acid to form dehydro-ascorbic acid and diketogluconic acid and the development of the yellow-orange colored composite was measured at 520 nm and defined as µg/mg protein [26]. Glutathione peroxidase (GPx, EC.1.11.1.9) activity was assessed using the method of Rotruck et al., which is based on the reduction of hydrogen peroxide by GSH for 5 min and the values are expressed as µmoles of GSH utilized/min/mg protein [27]. Glutathione-S-transferase (GST) activity was measured by using the method of Habig et al., which is based on the conjugation of the thiol group of glutathione with the 1-chloro, 2-4dinitrobenzene (CDNB) and the values are expressed as µmol of CDNB-GSH conjugate formed min/mg protein [28]. Glutathione reductase (GR) activity was measured by the method of Carlberg and Mannervick: Based on the reduction of glutathione disulfide to reduced glutathione, one unit of enzyme activity is defined as the nmoles of NADPH consumed/min/mg protein [29]. Cytochrome p450 and cytochrome b5 activity were measured according to the method proposed by Omura and Sato, the formation of carbon monoxide (CO) adduct reduced cytochrome p450 with CO, and the spectral difference between reduced and oxidized cytochrome b5 measured respectively [30].

### 2.10. Western Blot Analysis

According to the manufacturer’s instruction of protein isolation kit, total proteins were extracted from the buccal tissues of control and experimental groups. Each protein (50 mg) samples were separated through SDS-PAGE and then transferred to PVDF membranes by electrophoretically. The membrane was blocked with 5% nonfat dry milk for 2 h to block unspecific binding sites. The membrane was kept overnight incubation with 1:1000 dilutions of primary monoclonal antibodies Mutant p53 (catalogue No: ab32049; Abcam, UK), Bax, Bcl-2, cleaved caspase 3, cyclin-D1 and β-actin at 4 °C and detected with horseradish peroxidase-conjugated secondary antibody for 1 h. Finally, the transferred protein bands were visualized using enhanced chemiluminescence reagents and quantitated by ImageJ, a public domain Java image processing software (Wayne Rasband, NIH, Bethesda, MD, USA).

### 2.11. Statistical Analysis

Data were expressed as mean ± standard deviation. Statistical differences compared between treated groups and the untreated group were analyzed by one-way analysis of variance (ANOVA) and followed by Turkey HSD with IBM SPSS version 23.0 (SPSS Inc., NY, USA).

## 3. Results

### 3.1. Physiochemical Analysis of Polydatin Loaded Nanoparticles for the Determination of Size, Potential, and Morphological Features

**Figure 1:**
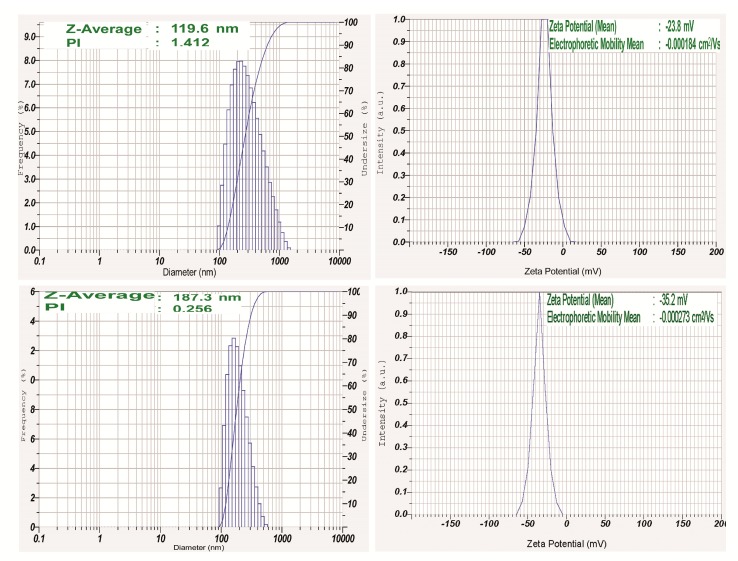
Dynamic light scattering (DLS) analysis (mean particle size, polydispersity index and Zeta potential) of poly-lactic-co-glycolic acid nanoparticles (PLGA-NPs) and POL-PLGA-NPs. POL-PLGA-NPs has 187.3 (average size), 0.256 (PDI index), and −23.8 mV of Zeta potential.

Size and shape of the nanoparticles are a key factor in designing of drug delivery systems. The high surface–volume ratios of smaller sized nanoparticles efficiently interact with active compounds and polymers which ultimately enhance the therapeutically efficacy of the drug. In this study, DLS study was undertaken to ascertain the particle size, distribution, polydispersity index, and the potential of the fabricated nanoparticles (Figure 1). It was seen that the average size of biosynthesized PLGA-NPs was 119.6 nm (PDI index: 1.412) and POL-PLGA-NPs was 187.3 nm (PDI index: 0.256). Surface charge of PLGA-NPs was found to be −35.2 mV and POL-PLGA-NPs was −23.8 mV.

**Figure 2:**
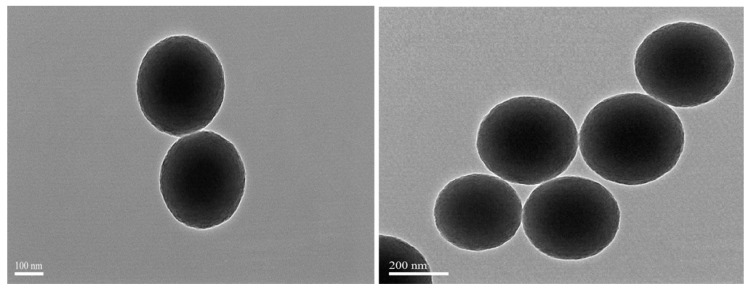
Transmission electron microphotograph of POL-PLGA-NPs. It shows the smooth surface without agglomeration and NPs appeared spherical in shape, with an average size of 105 to 200 nm.

**Figure 3:**
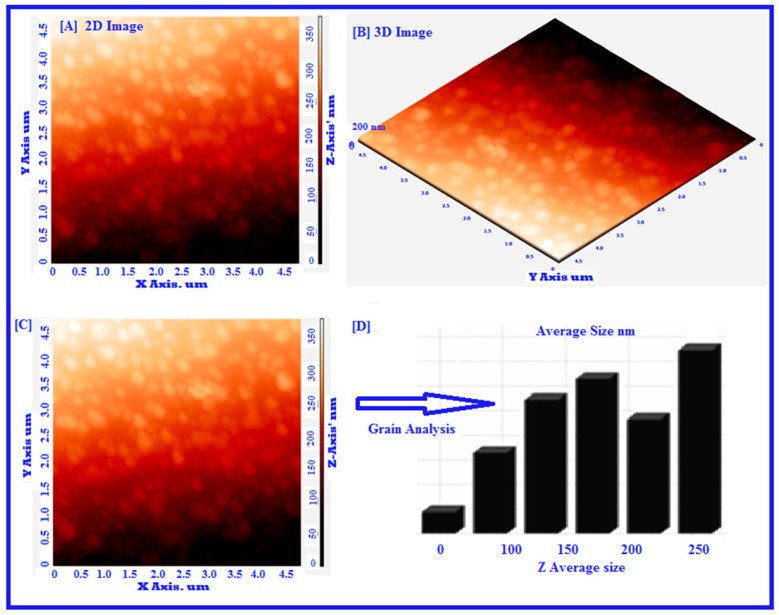
Atomic force microscopy analysis of green synthesized POL-PLGA-NPs. The results disclose that biosynthesized NPs appears to be spherical in shape by the seeing the nanoparticle’s topology and morphology of 2D image (**A**). Nova-Px 3.2.0.rev soft ware provided by NT-MDT was used to detect the grain size of the AFM image. An analysis of the results reveals that the NPs are varied in size that is 150 nm ± 10 nm to 200 nm ± 10 nm, we have also carried out grain analysis of the AFM 3D image using Nova-Px 3.2.0.rev software (**B**). Whereas the average size of the grains was found to be 160 ± 10 nm nm by using grain analysis (**C**&**D**).

TEM and SEM analysis were investigated to find out the surface morphology of synthesized nanoparticles. TEM images revealed that the smooth surface without agglomeration and fabricated nanoparticles appeared spherical in shape, with the average size of the particles ranging from 105 to 200 nm (Figure 2). Figure 3 showed the analysis of surface morphology and size distribution of synthesized POL-PLGA-NPs using atomic force microscopy. The results indicate that the NPs are spherical in shape and the size distribution of nanoparticles is between 120 to 210 nm (Figure 3A–D). The results are similar that of Particle size analysis and TEM analysis.

### 3.2. Elemental Analysis of FTIR and XRD Analysis

**Figure 4:**
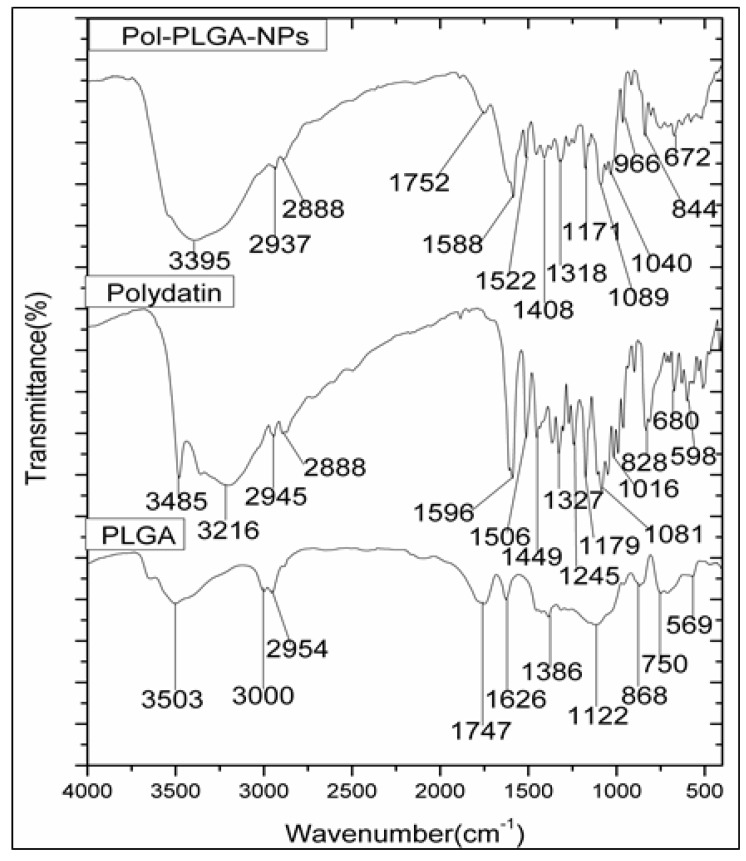
FTIR spectrum of polydatin, PLGA and POL-PLGA-NPs. FTIR spectrum of POL-PLGA-NPs showing the peaks of 3503, 3485 cm^−1^ shift to a lower frequency at 3395 cm^−1^ due to the encapsulation of PLGA.

Fourier-transform infrared spectroscopy (FTIR) analysis was conducted to identify the functional group analysis of Polydatin, PLGA and POL-PLGA-NPs (Figure 4). FTIR spectrum of polydatin was observed at 3485 cm^−1^ (O–H stretching), 2945 and 2888 cm^−1^ (C–H stretching), 1596 cm^−1^ (C=C stretching), and 11797 1081 cm^−1^ (C–O stretching), and intense peaks at 1506 cm^−1^ and 1449 cm^−1^ due to C-H bending, and 1327 cm^−1^ due to O-H bending for alcohol. Moreover, FTIR spectrum of PLGA showed distinct peaks at 3503 cm^−1^ (O–H stretching for acid group), 3000 and 2954 cm^−1^ (C-H stretching), 1747 cm^−1^ due to C=O stretching for carbonyl group), 1626 cm^−1^ (alkyl C=C stretching), 1386 cm^−1^ (O-H bending), 1122 cm^−1^ (C–OH stretching), 868 and 750 cm^−1^(C–H bending). On the other hand, the FTIR spectrum of POL-PLGA-NPs showed 3503 and 3485 cm^−1^ shifted to a lower frequency at 3395 cm^−1^ due to the encapsulation of polymer. The sharp peaks at 1747 and 1596 cm^−1^ were reduced to 1752 and 1588 cm^−1^ due to carbonyl groups. It was seen that all the characteristic peaks of polydatin and PLGA are visible in polydatin loaded PLGA nanoparticles.

**Figure 5:**
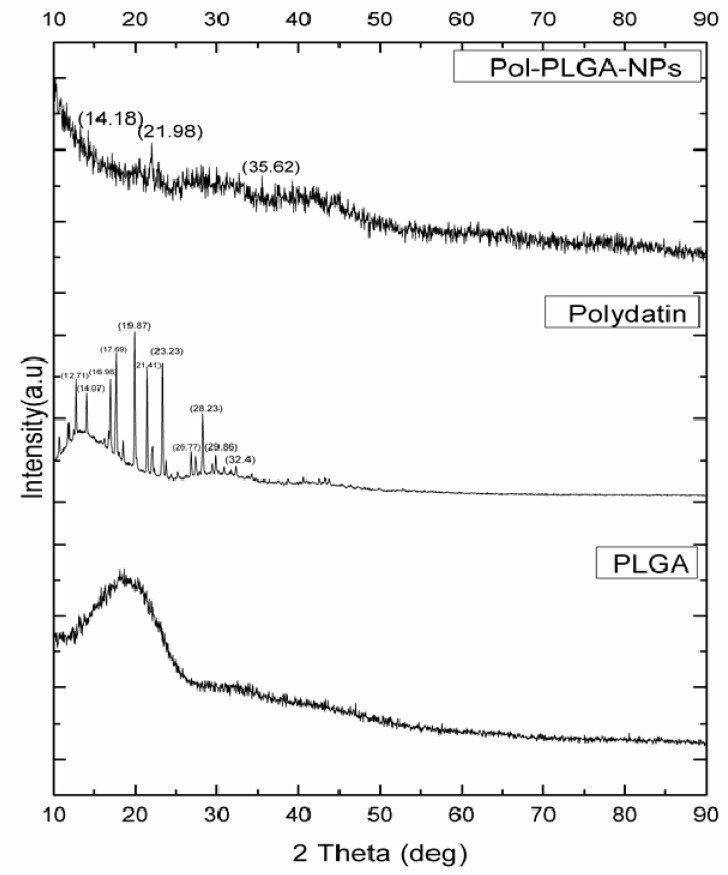
XRD pattern of polydatin, PLGA, and POL-PLGA-NPs. POL-PLGA-NPs showings peaks in 14.18°, 21.98°, and 35.62° indicates the entrapment of polydatin in synthesized PLGA nanoparticles.

The XRD pattern of polydatin clearly showed many intense and sharp peaks at 2θ at 12.71°, 14.07°, 16,98°, 17.69°, 19.87°, 21.41°, 23.23°, 26.77°, 28.23°, 29.86°, and 32.04°, which suggested its crystalline nature [29] as it has strong crystalline peaks. PLGA exhibits amorphous nature due to the presence of hump peaks. Besides, POL-PLGA-NPs showed peaks at 14.18°, 21.98°, and 35.62°, clearly indicating that the drug entrapped in nanoparticles and has amorphous nature (Figure 5).

### 3.3. Encapsulation Efficiency, Drug Loading, and Drug Releasing Profile of POL-PLGA-NPs

**Figure 6:**
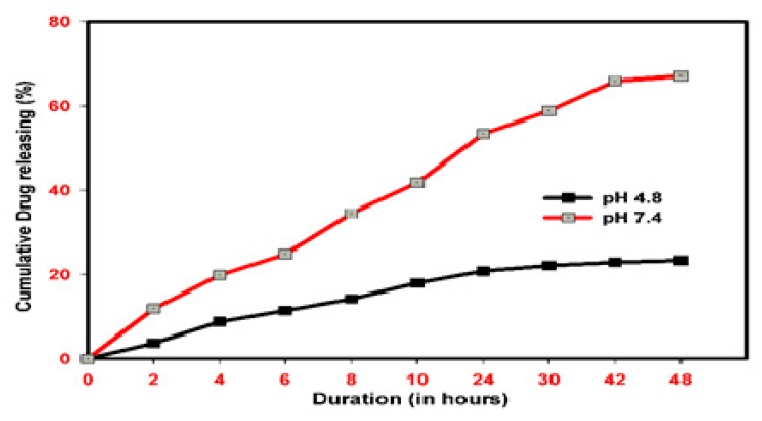
In vitro release pattern of polydatin loaded PLGA nanoparticles. Burst and fast releasing pattern were recorded in pH 4.8, Almost 68% polydatin was released from the nanoformulation.

Table 1 shows the drug loading and encapsulation efficiency of POL-PLGA-NPs with different concentrations of POL., i.e., at 1, 3, and 5 mg/mL. The nanoparticles with 5 mg/mL of POL showed remarkable drug loading and encapsulation efficiency of 8.71 ± 0.74% and 94.52 ± 9.23%, respectively. As shown in Figure 6, the releasing patterns of POL-PLGA-NPs reveals that the fabricated nanoparticle has the pH-independent drug releasing profile. Burst and fast releasing patterns were recorded at pH 5.5. Nearly 50% of polydatin was released in the initial 2 h, and later the release was very slow. A maximum of 68% polydatin was released from the nanoformulation of POL-PLGA-NPs at 48 h. No more release was recorded after that. Also, the sustained drug releasing profile was recorded at pH 7.4. About 16% of the drug was released in an initial period of 2 h, and only 23% drug was released in 48 h.

### 3.4. POL-PLGA-NPs Suppress the DMBA Induced Neoplastic Changes

Body weight changes, tumor formation, and multiplicity POL-PLGA-NPs treated groups showed a significant gradual increase in body weight (149.97 ± 8.61, 151.16 ± 10.81, and 168.33 ± 14.17). The decreased body weight was evident in carcinogen-alone treated animals (112.73 ± 4.21), whereas the mean body weight of control animals was 192.45 ± 7.17 (Table 2). The administration of POL-PLGA-NPs did not show any clinical sign of toxicity, thus confirming the non-toxic effects of biosynthesized POL-PLGA-NPs and their dosage levels. The site-specific carcinogen DMBA caused 100% of tumor incidence in all carcinogens-alone painted animals, which shows the potential of the carcinogen (Table 2). The total number of tumors and number of tumors and tumor-bearing animals was significantly high in group 2 among all DMBA treated animals (*p* < 0.05). In addition, the high tumor volume indicates the aggressiveness of the disease. Administration of POL-PLGA-NPs to DMBA treated animals (groups 3–5) showed a remarkable decrease in tumor volume and percentage incidence. There were no tumors found in the control (group 1) and drug control (group 6) animals. DMBA-alone painted tumor-bearing animals (group 2) showed the histological characterization of tumor, such as severity in keratosis, hyperplasia, dysplasia, and moderate levels of squamous cell carcinoma, whereas the treatment with POL-PLGA-NPs reduced the tumor histological characteristics from severe to moderate, and inhibited the formation of squamous cell carcinoma. No histological abnormalities were found in the control and drug control animals (groups 1 and 6).

### 3.5. POL-PLGA-NPs Enhances the Lipid Peroxidative Byproducts

**Figure 7:**
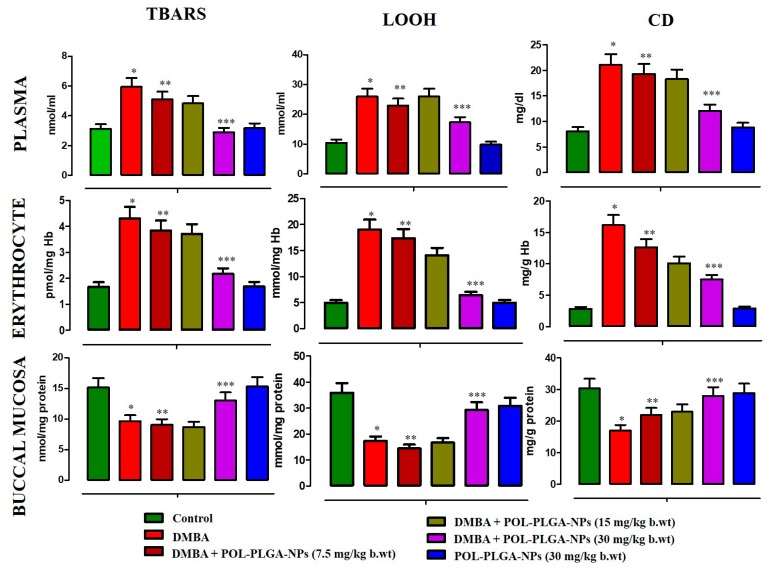
Effects of POL-PLGA-NPs on 7, 12-dimethyl benzyl anthracene (DMBA) -induced changes in the activity of thiobarbituric acid reactive substances (TBARS), Lipid hydroperoxides (LOOH), and CD. Values are expressed as mean ± SD (*n* = 6). * significantly differ from control group (*p* < 0.05), ** Significantly differ from DMBA group (*p* < 0.001), *** significantly differ from DMBA group (*p* < 0.05) (One-way ANOVA).

The levels of TBARS, LOOH, and CD in circulation and buccal mucosa of DMBA-treated and control groups are shown in Figure 7. Exposure to carcinogen exhibited significant increase (*p* < 0.05) in the levels of TBARS, LOOH, and CD at the end of 16 weeks. On supplementation of POL-PLGA-NPs to DMBA exposed animals revealed significantly reduced levels (*p* < 0.05) of lipid peroxidative byproducts levels.

### 3.6. Enzymic and Non Enzymic Antioxidant Status

**Figure 8:**
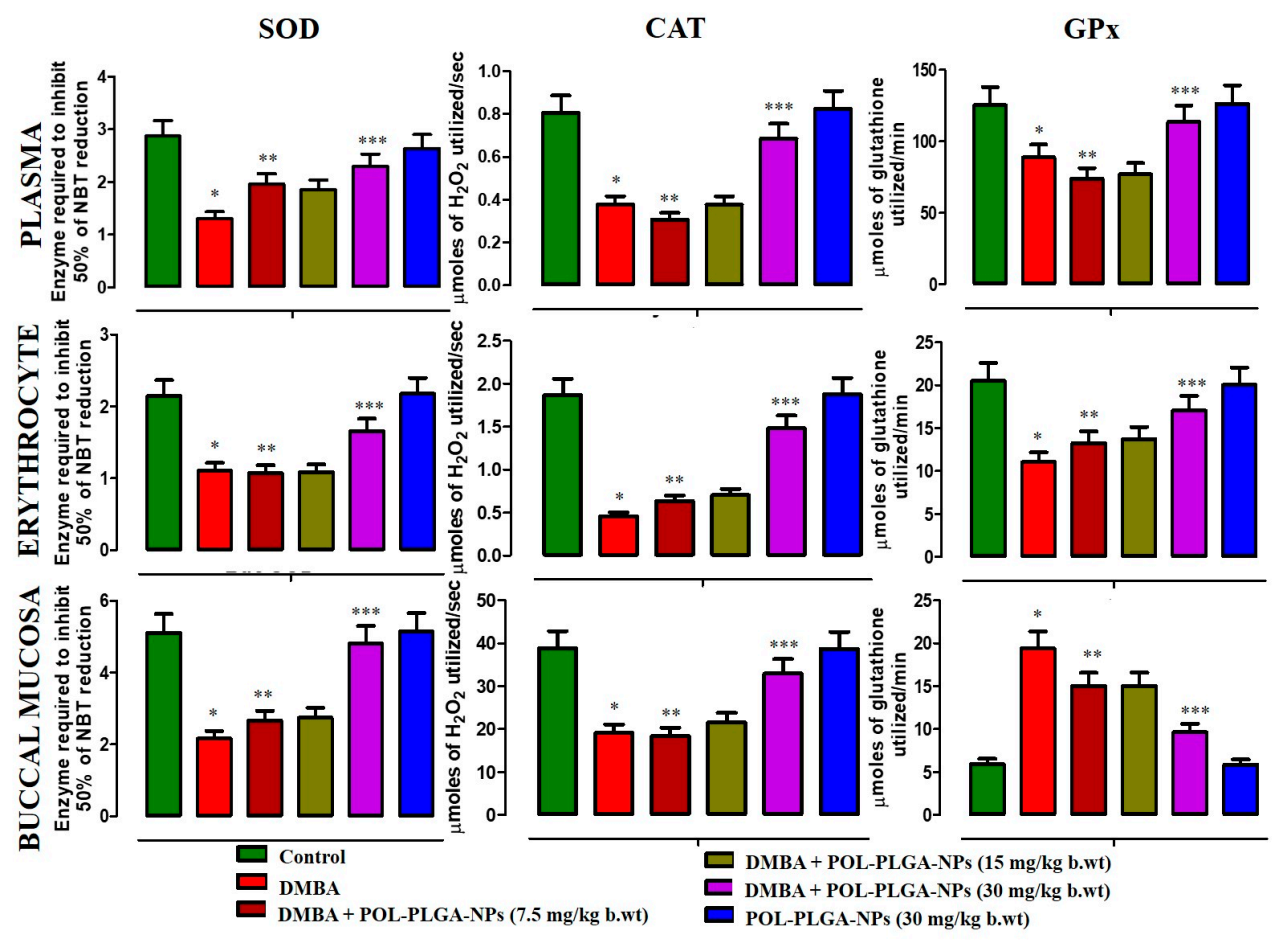
Effects of POL-PLGA-NPs on DMBA induced changes in the activity of enzymatic antioxidants. Values are expressed as mean ± SD (*n* = 6). * Significantly differ from control group (*p* < 0.05), ** Significantly differ from DMBA group (*p* < 0.001), *** significantly differ from DMBA group (*p* < 0.05).

Figure 8 presents the activities of enzymic antioxidant levels in circulation and buccal mucosa of control and DMBA-exposed animals. DMBA-alone painted animals show the reduced levels of enzymic antioxidants such as SOD, CAT, and GPx levels, whereas oral supplementation of POL-PLGA-NPs to DMBA-painted animals significantly improved (*p* < 0.05) the levels of above said antioxidants. There were no significant differences between control and drug control animals.

**Figure 9:**
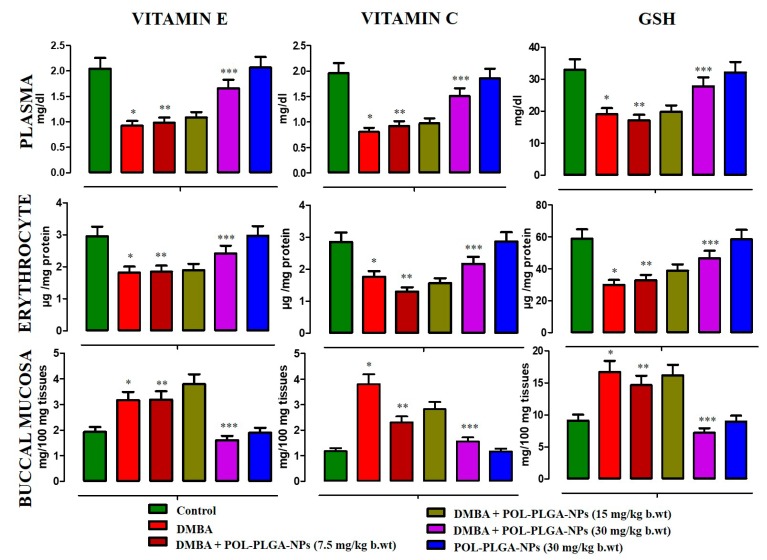
Effects of POL-PLGA-NPs on DMBA induced changes in the level of non-enzymatic antioxidants. Values are expressed as mean ± SD (*n* = 6). * Significantly differ from control group (*p* < 0.05), ** Significantly differ from DMBA group (*p* < 0.001), *** Significantly differ from DMBA group (*p* < 0.05) (Oneway ANOVA).

The levels of non-enzymic antioxidants in the circulation and buccal mucosa of experimental animals were shown in Figure 9. A significant reduction was observed in the levels of non-enzymic antioxidants such as Vitamins E, C, and reduced glutathione in carcinogen-treated unsupplemented animals. Upon treatment with POL-PLGA-NPs significantly (*p* < 0.05) increases the levels of those non-enzymic antioxidants to bring back near control values.

### 3.7. Xenobiotic Metabolizing Enzymes

**Figure 10:**
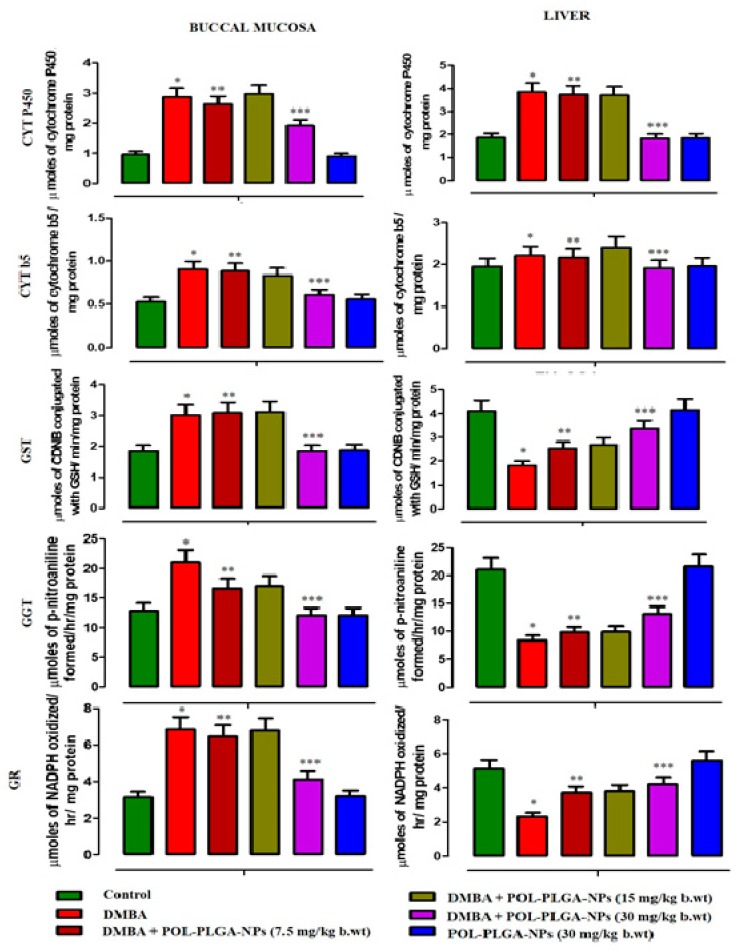
Modulating effects of POL-PLGA-NPs on DMBA induced Xenobiotic metabolizing enzymes. Values are expressed as mean ± SD (*n* = 6). * significantly differ from control group (*p* < 0.05), ** significantly differ from DMBA group (*p* < 0.001), *** significantly differ from DMBA group (*p* < 0.05) (One-way ANOVA).

The xenobiotic metabolizing enzymes levels of control and DMBA painted animals were shown in Figure 10. Carcinogen alone expose animal showed a considerable increase in Phase I and Phase II metabolizing enzymes such as Cyt p450, Cyt b5, GST, GGT, and GR activities. POL-PLGA-NPs supplementation to DMBA painted animals reduces the levels of those phases I and II enzymes on a dose-dependent basis, which was more pronounced in POL-PLGA-NPs (30 mg/kg b.wt).

### 3.8. Effect of POL-PLGA-NPs on the Histopathological Features of the DMBA Induced Buccal Pouch Carcinogenesis

**Figure 11:**
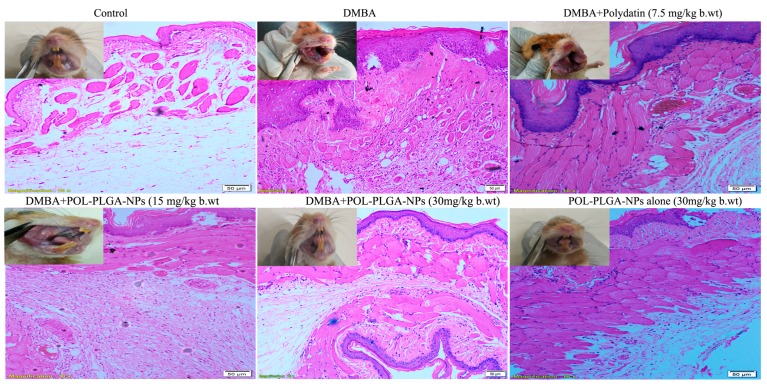
Histopathological analysis of buccal tissue of control and experimental animals (10x). Control and POL-PLGA-NPs showing normal architecture. DMBA alone treated sections showing a well-defined squamous cell carcinoma with hyper chromatic nuclei containing epithelial and keratin pearls. DMBA+POL-PLGA-NPs showing a mild to moderate dysplasia and hyperplasia.

The histopathological evaluation of the buccal tissues of control, carcinogen-alone and POL-PLGA-NPs-treated animals are presented in Figure 11. At the end of 16 weeks, squamous cell carcinoma was evident in the carcinogen-alone exposed group initiated with DMBA. Hyperkeratosis, along with hyperplasia and dysplasia was also observed in DMBA-alone exposed animals. POL-PLGA-NPs-treated DMBA-painted animals displayed mild keratosis as well as mild hyperplasia and dysplasia.

### 3.9. Effect of POL-PLGA-NPs on Apoptotic and Proliferative Marker Expressions in DMBA Induced Buccal Pouch Carcinogenesis

**Figure 12:**
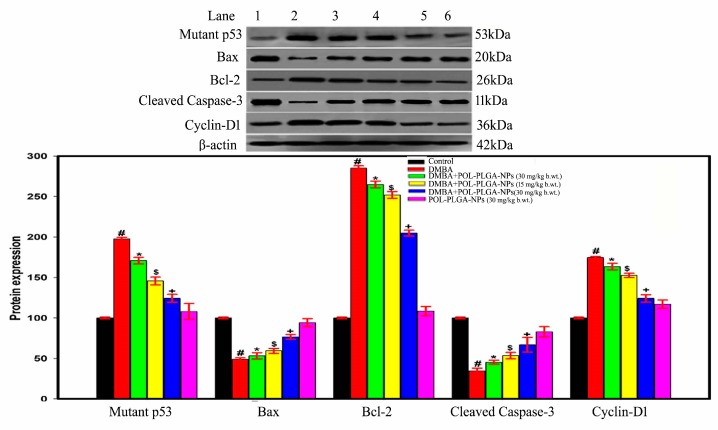
Immunoblot analysis of mutant p53, bax, Bcl-2, cleaved caspase-3, and cyclin D1 in control an experimental animal. 1-Control; 2-DMB; 3-DMBA+POL-PLGA-NPs (7.5 mg/kg b.wt.); 4-DMBA +POL-PLGA-NPs (15 mg/kg b.wt.); 5-DMBA +POL-PLGA-NPs (30 mg/kg b.wt.); 6-POL-PLGA-NP alone (30 mg/kg b.wt.). POL-PLGA-NPs enhance the apoptotic mediators such as Bax, cleaved caspase-3 and inhibit p53, Bcl-2 and cyclin-D1 expressions in a dose-dependent manner. Bar diagram represents the proteins expression; each bar represents the mean ± SD of three independent analysis.

The role of POL-PLGA-NPs and/or DMBA-mediated protein expression of apoptotic and proliferative markers were studied by western blotting analysis (Figure 12). The proapoptotic marker Bax, cleaved caspase-3 was highly down regulated and proliferative marker mutant p53, Bcl-2, and cyclin-D1 were extensively over expressed during the exposure of DMBA in rat buccal pouch. On the other hand, the delivery of POL-PLGA-NPs induces apoptotic mediators such as Bax, cleaved caspase-3 and inhibits DMBA induced mutant p53, Bcl-2, and cyclin-D1 expressions in a dose-dependent manner. We noticed that POL-PLGA-NPs (30 mg/kg.b.wt) treatment was a more efficiently remarkable activity in DMBA-exposed hamsters, whereas POL-PLGA-NPs (30 mg/kg.b.wt) alone produced no toxicity.

## 4. Discussion

Ploydatin has strong antioxidant activities. Owing to conjugated double bonds in its molecular structure, it shows many beneficial pharmacological activities such as improving learning and memory, lipid lowering, and extending lifespan. Conjugated compounds can absorb electrons and form adducts with oxygen species (e.g., epoxides, diols and other structures), and thus behave like antioxidants. Although they are antioxidants as well, they form adducts with protein SH groups, thereby activating Nrf-2 antioxidant signaling pathways [31]. Through hydrophobic stacking and hydrogen bonds, polydatin can interact with neurotensin (NT). The polyphenol–protein complexes seem to affect NT metabolism and diminish the NT-induced metabolic activation of colon carcinoma cells [32]. Mikulski and Molski (2010) reported that the presence of 4′-OH group is primarily responsible for the antioxidant capacity [33]. However, to improve the meditative potential of phytocompounds, polymer-based nanoparticles can be attainable feasible approach to improve the biocompatibility and shield against digestive enzymes and pH changes.

Polymer based nanoparticles have attracted the attention of the modern scientific community due to their fascinating applications in biomedical sciences. Scientific evidences have demonstrated that PLGA-based nanoparticles are capable of inducing apoptosis and arresting the cell proliferation in cancerous conditions acts as a carrier molecule to enhance the stability and pharmacological activity of polydatin [34]. Based on this information, the oil/water emulsion method was commonly used in the preparation of nanocarrier with therapeutic agent embedded with hydrophobic or polymeric lattice. This method allows for rapid access of nanospheres or nanocapsules in large quantity and scale up pharmaceuticals industries. Based on this literature, the oil/water emulsion method was used for fabrication of polydatin loaded PLGA nanoparticles (POL-PLGA-NPs). There was a strong ionic interaction between the polydatin and PLGA facilitate the formation of nano-sized particles with the help of stirring and sonication. In the study, average diameter of the fabricated POL-PLGA-NPs was found to be 187.3 nm. In the study, the average diameter of the fabricated POL-PLGA-NPs was found to be 187.3 nm. Due to the presence of terminal carboxyl groups in the PLGA ensures the negative potential, which again ensures abiding stability and avoids particle aggregation. This result correlates with the previous findings that nanoparticles with the average size of 400–600 nm are able to penetrate the endothelial gap of the tumor tissue [35]. The findings of the TEM analysis and 3D analysis of AFM studies were confirmed that the synthesized nanoparticles were typically uniform and spherical shaped nanoparticles with an average size range of 144 nm to 200 nm. Similarly, Lozano et al. reported that the nanoencapsulated quercetin was found to be spherical in shape with an average from 90 nm to 165 nm [36]. The particle size recognized from TEM and AFM analysis strongly supports the findings of DLS analysis.

X-ray diffraction (XRD) analysis is a non-destructive technique generally used to scrutinize the crystallinity and physical nature of the nanoparticles. XRD patterns of polydatin, PLGA, and POL-PLGA-NPs were acquired and compared the significant differences in the molecular state of the nanoformulation. A hump peaks at 20° (2θ), which is pinpointing of the amorphous nature of PLGA. Whereas a sharp peak observed at 19.87°, 23.23°, and 28.23° indicating the crystalline nature of the polydatin. Upon the integration of polydatin into PLGA nanoformulation showed the less intensity of peaks at 21.98° clearly indicates the amorphization nature. Earlier studies also documented that XRD pattern of the encapsulation NPs were exhibited less intensity of peaks when compared with plant-based phytochemicals, which clearly indicate the reduction in the crystallity form the nanoparticles. Similar observation was made our study. In the present study, FTIR patterns of POL-PLGA-NPs strongly suggest that PLGA nanoparticles were successfully encapsulated with a bioactive molecule of polydatin by oil/water emulsion method. The major peaks at 3395 and 1588 cm^−1^ became wider and flatter; indicating that hydrogen bond was enhanced, that there were no loss of functional groups in nanoformulation, and that the crystalline structure was imported to the PLGA nanoparticles.

Encapsulation and loading efficiency of nanoparticles is considered to be one of the critical qualities that improve bioavailability of the drug. The particles having higher loading ability form inefficient delivery systems. The nanoparticles with 5 mg/mL of POL showed remarkable drug loading and encapsulation efficiency of 10.71 ± 0.74% and 96.54 ± 8.03%, respectively. It was revealed that the drug releasing profile of POL-PLGA-NPs was directly equivalent to the concentration of POL. The loaded formulation of POL significantly enhanced the drug loading efficiency of the nanoparticles due to the strong hydrophobic interaction of PLGA. This speculation is in line with numerous studies which testified that PLGA-mediated nanoparticles enhance the drug loading efficiency [37,38]. The findings of the in vitro drug releasing pattern of POL-PLGA-NPs revealed that it is minimally released in normal healthy cells and tissue (pH 7.4), whereas at pH 4.8, the formulated polydatin may attack the tumor tissue and ultimately enter inside the cancer cells to selectively kill the cancer cells due to its nano size and high negative potential.

DMBA is classified as a polycyclic aromatic hydrocarbon. It is an indirect carcinogen that needs metabolic activation to yield an ultimate carcinogen. Initially, the oxidation reaction converts DMBA to DMBA-3,4-epoxide by phase I xenobiotic metabolizing enzymes, especially cytochrome p450 [39]. The epoxide hydratase, another phase I enzyme, converts the epoxide to DMBA-3, 4- diol, theproximate carcinogen. Following a series of oxidation steps by cytochrome leads to synthesis of DMBA-3,4 dioll,2-epoxide, the ultimate carcinogen, which reacts with purine molecules to form DNA adducts [40].

Weight loss is a common characteristic in tumor-bearing animals. DMBA-alone painted animals show a drastic weight reduction along with reduced growth rate, showing the alteration in body metabolism which breaks down the proteins and lipids. In particular, glucose metabolism in cancerous-stage whole-body glucose turnover rate may increase, which increases hepatic glucose synthesis, or gluconeogenesis, from substrates derived from proteolysis and lipolysis [41]. The site-specific carcinogen DMBA induces multiple tumors in buccal tissue with malignant features. The tumor incidence was 100% in carcinogen-alone animals, validating the potency of the carcinogen, and its characteristics revealing the aggressiveness of the disease. Intragastric administrations of POL-PLGA-NPs at the different dosages inhibit the formation of tumors and prevent the tumor growth.

The number and percentage of tumors also reduced in treatment with POL-PLGA-NPs. In particular, at 30 mg/kg b.w., the chemopreventive potential is realized either by preventing or inhibiting the formation of tumor. Martano et al. (2018) supported the use of polydatin in oral cancer prevention and/or as alimentary support associated with anti-tumoral therapy, which is evident from the present study [42]. The PLGA coated nanoparticles may penetrate epithelial cells to enter into the circulation, and accumulate inside the tumor to prevent further progression. Oral cancer was histopathologically confirmed as well differentiated squamous cell carcinoma. DMBA requires metabolic activation by cytochrome p450 to form diolepoxide and other ROS that are known to increase intracellular oxidation, causing severe damage to DNA, lipids and proteins, and thereby contribute to carcinogenesis [43].

ROS mediated oxidative stress has been implicated in the membrane lipid peroxidation, which include increased membrane fragility, decreased red cell fluidity, altered cell function, and structural integrity [44]. Several studies reported the relationship between ROS-mediated lipid peroxidation and several diseases, including oral cancer. The byproducts of lipid peroxidation, reactive aldehydes often form bioactive adducts with macromolecules that are important for the pathophysiology of living cells, thus simulating the impacts of reactive oxygen species (ROS) even in the lack of serious oxidative stress [45,46]. Blood can reflect the liability of the entire animal to oxidative circumstances and it is also a major target of oxy radical assault [47]. Free radicals released into circulation eventually cause hemolysis [48]. When there is an imbalance between prooxidants and antioxidants, it results in increased free radical production and excessive antioxidant consumption, which are the causative factors for oxidative damage [49]. The enhanced lipid peroxidation in the circulation of tumor-bearing animals reflect excessive free radical generation exacerbated by a decreased efficiency of the host antioxidant defense mechanisms. Tumor cells generate and release peroxides into the circulation which can subsequently oxidize GSH. Tumor cells also sequester antioxidants from circulation to promote tumor growth. This may be one of the reasons for the declined antioxidant status with enhanced lipid peroxidation in the circulation of the DMBA treated animals. Increased plasma TBARS observed in tumor-bearing animals are probably due to the overproduction and diffusion of lipid peroxidation byproducts from the damaged tissues with consequent leakage to the plasma. On supplementation with POL-PLGA-NPs, the levels of plasma and erythrocytes TBARS in DMBA-treated animals significantly decreased. This suggests that POL-PLGA-NPs have anti-lipid peroxidative potential during oral carcinogenesis. Numerous studies have demonstrated the increased lipid peroxidation and declining antioxidant status in experimental oral carcinogenesis [50]. On the other hand, tumor tissue has the ability to prevent lipid peroxidation through the highly evolved protective mechanisms so that rapid cell proliferation can occur [51]. Cancer cells are known to acquire certain characteristics that benefit proliferation [52] and they tend to proliferate faster when lipid peroxidation is low. Moreover, malignant tissues are less susceptible and more resistant to free radical attack and hence lipid peroxidation is less intense [53]. Thus, we observed decreased lipid peroxidative that rapid progression of tumor. However, the administration of POL-PLGA-NPs to tumor-bearing animals brings back the lipid peroxidative byproduct levels to near control.

The enzymic antioxidants such as SOD, CAT, and GPx function as the front line of defense against oxidative stress by virtue of their ability to catalyze the disproportionation reactions of their substrate free radicals that are spontaneously generated by in vivo oxidative phosphorylation, cytochrome p450 metabolism, and inflammatory processes [54]. Catalase is a catalyst that changes H_2_O_2_ to nonpartisan items, O_2_, and H_2_O. GPx is an initiated protein that acts against oxidative damage, and this requires glutathione as a cofactor. It catalyzes the oxidation of GSH to GSSG to the detriment of H_2_O [55]. In the carcinogen-treated animals, the activities of SOD, CAT, and GPx were reduced, which shows the high utilization of endogenous antioxidants and need of more to scavenge the radicals induced by DMBA.

Administration of POL-PLGA-NPs to DMBA-exposed animals reduces the scavenging activities of antioxidants, thereby reducing the oxidative stress. In addition to the above, DMBA-treated animals lessened the levels of Vitamins E and C in the blood and buccal mucosa, and the exercises of these catalysts were impeded because of rehashed exacerbation by the carcinogen. On supplementation of POL-PLGA-NPs, the levels of Vitamins E and C were recovered to near control. In contrast, GSH levels were upheld in the carcinogen-exposed animals, due to uncontrolled proliferation of tumor cells; however, the POL-PLGA-NPs minimize the utilization of glutathione and inhibit the tumor growth process. The cytochrome p450 (oxidizing phase I metabolizing enzymes) is a group of enzymes playing a central role in oxidative metabolic activity [56]. The metabolic activation of DMBA produces diol epoxides, and various ROS, RNS are known to cause damages to lipids, protein, and nucleic acids [57]. Supplementation of POL-PLGA-NPs on DMBA treated animals bring back the phase I and II enzymes levels to near control in buccal mucosa and liver tissues. This finding suggests that POL-PLGA-NPs play a crucial role in the detoxification of DMBA.

Inductions of oral carcinogenesis have been associated with the failure of apoptosis and subsequent activation of proliferation. Bax, Bcl-2, and caspases are involved in the proapoptosis process. Cyclin-D1 and mutant-p53 are deeply involved in proliferation. These apoptotic and proliferative markers are substantially analyzed by western blotting. The in active form of caspase-3 (pro enzyme), is cleaved at an aspartate residue to yield a p12 and p17 subunit to form the active caspase-3 enzyme (cleaved caspase-3) that is responsible for morphological and biochemical changes in apoptosis and is useful in scoring the apoptotic index. Aberrant caspase-3 protein expression has been extensively studied. On this basis, we investigated the expressions of cleaved caspase-3 in tumorigenesis. The inhibition of cell proliferation was measured by evaluating the protein expression levels of Bcl-2. Bcl-2 is an integral membrane protein located mainly on the outer membrane of mitochondria. Overexpression of Bcl-2 prevents cells from undergoing apoptosis in response to a variety of stimuli. Cytosolic cytochrome c is necessary for the initiation of the apoptotic program, suggesting a possible connection between Bcl-2 and cytochrome c, which is normally located in the mitochondrial intermembrane space. Cells undergoing apoptosis were found to have an elevation of cytochrome c in the cytosol and a corresponding decrease in the mitochondria. Overexpression of Bcl-2 prevented the efflux of cytochrome c from the mitochondria and the initiation of apoptosis. Thus, one possible role of Bcl-2 in prevention of apoptosis is to block cytochrome c release from mitochondria. Moreover, dysregulation of cell death genes leading to overexpression of Bcl-2 or reduction in Bax expression, for example, would alter the Bcl-2: Bax ratio which is considered to be anticarcinogenic, and vice versa [58]. As expected, the levels of Bcl-2 in carcinogen treated animals were elevated and treatment with POL-PLGA-NPs to tumor-bearing animals reduces the Bcl-2 protein level. On the other hand, Bax protein levels were increased in tumor-bearing POL-PLGA-NPs-treated an animal, which shows the anticarcinogenic potential of POL-PLGA-NPs. Moreover, POL-PLGA-NPs treatment induces apoptotis through the over-expression of cleaved caspase-3 and inhibits DMBA-induced mutant p53 and cyclin-D1 expressions in a dose-dependent manner. We noticed that POL-PLGA-NPs (30 mg/kg.b.wt) treatment more efficient and remarkable in DMBA-exposed rats. These results are closely correlated with the activity of detoxification enzymes. Previously, metformin-encapsulated PLGA-PEG nanoparticles induced apoptosis by the expression of p53, Bax and caspase-3 in ovarian cancer [59]. It was concluded that the nanoformulation of polydatin may enhance the mitochondrial-mediated apoptotic mechanism in DMBA-treated hamsters.

## 5. Conclusions

**Scheme 1:**
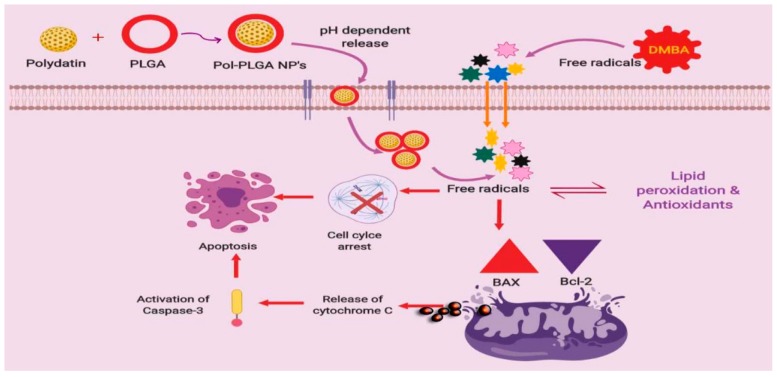
Proposed mechanism involved in the polydatin loaded PLGA nanoparticles enhance the apoptosis and inhibit the cell proliferation in DMBA induced experimental carcinogenesis.

In conclusion, overall findings proposed that the green based POL-PLGA-NPs formulation inhibited the progression of tumor and its growth during DMBA initiated carcinogenesis in golden Syrian hamsters. In addition, the synthesized POL-PLGA-NPs shows strong antioxidant activities and reduces the tissue lipid peroxidation and spares the function of xenobiotic metabolizing enzymes, thereby shows potent chemopreventive efficacy evidenced by pathological reports (Scheme 1). These findings hopefully provide new insights of nanochemopreventive potential of POL-PLGA-NPs. This might pave the way for next generation of nano drug, which might be less expensive and with minimum side effects. Further studies on the bioavailability of the synthesized POL-PLGA-NPs are warranted in experimental animals. In the future, POL-PLGA-NPs may be useful for cancer therapies as an individual drug or in combination with other drugs.

## Figures and Tables

**Table 1:** Loading and encapsulation efficiency of polydatin loaded nanoparticles.

Concentration of Polydatin	1 mg/mL	3 mg/mL	5 mg/mL
Loading efficiency (%)	3.81 ± 0.25	7.29 ± 0.63	10.71 ± 0.74
Encapsulation efficiency (%)	22.78 ± 1.37	83.15 ± 6.22	96.54 ± 8.03

Values are expressed as the mean ± SD (*n* = 3).

**Table 2:** Body weight, incidence of oral neoplasm and histological features in POL-PLGA-NPs treated control and experimental animals.

Parameters	Control	DMBA	DMBA+ POL-PLGA-NPs (7.5 mg/kg b.wt.)	DMBA+ POL-PLGA-NPs (15 mg/kg b.wt.)	DMBA+ POL-PLGA-NPs (30 mg/kg b.wt.)	POL-PLGA-NP alone (30 mg/kg b.wt.)
Initial Bodyweight (g)	125.24 ± 6.47 ^a^	120.15 ± 3.03 ^b^	131.15 ± 9.03 ^b^	130.24 ± 12.16 ^c^	126.82 ± 8.04 ^e^	130.47 ± 7.95 ^a^
Final Bodyweight (g)	193.45 ± 7.17 ^a^	136.73 ± 9.01 ^b^	149.97 ± 8.61 ^c^	151.16 ± 9.81 ^d^	168.33 ± 9.17 ^e^	183.12 ± 8.07 ^a^
Weight Gain(g)	68.21 ± 6.72 ^a^	16.54 ± 1.72 ^b^	18.82 ± 5.72 ^c^	20.92 ± 6.74 ^d^	43.51 ± 6.38 ^e^	52.65 ± 5.21 ^a^
Tumor Incidence	-	100	80	68	20	-
Total number of tumors/animals	-	12/(6)	10/(6)	7/(6)	2/(6)	-
Tumor Burden	-	2024.76 ± 82.6 *	1586.2 ± 62.45	942.61.54.83	105.73 ± 7.11 ***	
Tumor Volume	-	168.73 ± 6.43 *	158.6 ± 5.84	134.3 ± 4.81	52.86 ± 1.33 ***	-
Keratosis	Not observed	Severe	Moderate	Moderate	Mild	Not observed
Hyperplasia	Not observed	Severe	Moderate	Moderate	Mild	Not observed
Dysplasia	Not observed	Severe	Moderate	Moderate	Mild	Not observed
Squamous cell carcinoma	Not observed	Well differentiated	Severe	Moderate	Mild	Not observed

Values are expressed as the mean ± SD for 6 hamsters in each group. * Significantly differ from control group (*p* < 0.05), *** Significantly differ from DMBA group (*p* < 0.05) (Oneway ANOVA). Groups not sharing a common superscript letter (a–e) differ significantly at *p* < 0.05 (Oneway ANOVA)
